# Exclusive breastfeeding during the 40-day rest period and at six months in Lebanon: a cross-sectional study

**DOI:** 10.1186/s13006-020-00289-6

**Published:** 2020-05-19

**Authors:** Rana F. Chehab, Lara Nasreddine, Racha Zgheib, Michele R. Forman

**Affiliations:** 1 Department of Nutrition Science, Purdue University, West Lafayette, IN 47906 USA; 2 Department of Nutrition and Food Science, American University of Beirut, Beirut, Lebanon; 3 Inserm 1256 NGERE, Nancy, France

**Keywords:** Exclusive breastfeeding, 40-day rest period, Socio-economic status, C-section delivery, Lebanon

## Abstract

**Background:**

Exclusive breastfeeding is recommended for the first 6 months of life with well-established benefits to the mother and child. The traditional practice of the 40-day rest period helps establish and maintain exclusive breastfeeding. This study aims to estimate the prevalence and examine the factors associated with exclusive breastfeeding at 40 days and at 6 months in Lebanon.

**Methods:**

A cross-sectional survey was conducted in 2011–2012 as part of the “Early Life Nutrition and Health in Lebanon” study. A nationally representative sample of 1005 children aged five years or younger and their mothers was drawn from households using a stratified cluster sampling design. Trained nutritionists interviewed eligible mothers about sociodemographic characteristics of the household and maternal and child characteristics including infant feeding practices. Anthropometric measurements of the mother and child were collected. Multinomial logistic regression analysis was conducted to examine the characteristics associated with exclusive breastfeeding.

**Results:**

The prevalence of exclusive breastfeeding was 41.5% at 40 days and 12.3% at 6 months. Children in families with three or more children had higher odds of exclusive breastfeeding for 40 days (Adjusted Odds Ratio [AOR] 1.76, 95% Confidence Interval [CI] 1.19, 2.60). Children in families owning two or more cars had lower odds of exclusive breastfeeding for 40 days (AOR 0.45, 95% CI 0.24, 0.83) and at 6 months (AOR 0.32, 95% CI 0.14, 0.77). Similarly, children delivered via Caesarian section had lower odds of exclusive breastfeeding for 40 days (AOR 0.49, 95% CI 0.34, 0.71) and at 6 months (AOR 0.39, 95% CI 0.24, 0.65). The odds of exclusive breastfeeding for 6 months were lower among children of overweight (AOR 0.50, 95% CI 0.26, 0.95) or obese (AOR 0.56, 95% CI 0.32, 0.98) mothers.

**Conclusions:**

The association between higher socio-economic status, as reflected by car ownership, and C-section delivery with lower odds of exclusive breastfeeding persisted across the first 6 months in Lebanon. Future research should investigate the factors associated with exclusive breastfeeding in prospective cohort studies and help to better understand the cultural practice of the 40-day rest period in relation to breastfeeding.

## Background

Many societies in the Middle East observe a 40-day postpartum period of rest, seclusion, and ritual that helps establish and maintain breastfeeding [1, 2] and protects the mother and newborn from illnesses [3]. During this 40-day rest period, the mother often stays at home and receives help with household chores and congratulatory visits from related women and neighbors [3]. She is encouraged to eat a special diet rich in meat, poultry, soups, and other foods thought to be good for milk production [3]. Other than the cultural aspect, the 40-day rest period has religious underpinnings. For both Muslims and Christians, the 40 days following birth coincide with the period of vaginal discharge resulting from involution of the uterus [4–6]. Despite being closely linked to infant feeding practices, few studies have examined the 40-day rest period in relation to total and exclusive breastfeeding [1, 2].

The World Health Organization (WHO) recommends exclusive breastfeeding (EBF) for the first 6 months of life [7]. Despite these recommendations and the well-established benefits of breastfeeding, the proportion of mothers who EBF for 6 months in the Middle East is estimated at 20.5% (95% CI 14.5, 28.2) [8]. The prevalence of EBF for 6 months in specific countries of the Middle East ranges from 2% in Kuwait [9] to 56.4% in Iran [10]. In Lebanon, a small Middle Eastern country on the Mediterranean sea, the prevalence of EBF for 6 months was 10.1% in a national sample of mother-child dyads recruited from primary healthcare centers in 2004 [11]. More recently in 2016, the prevalence of EBF for 4–6 months was estimated at 16.5% among mothers of toddlers attending daycare centers [12].

None of the previous studies conducted in Lebanon focused on the 40-day rest period as a cultural practice for breastfeeding. Understanding the context-specific patterns and determinants of breastfeeding practices is essential to ensure successful promotion strategies [13]. Therefore, it is timely to place a lens on the 40-day rest period and to examine the factors associated with EBF in Lebanon. The objectives of this paper are to estimate the prevalence of EBF at 40 days and at 6 months, and to examine the sociodemographic, maternal, and child factors associated with EBF in a nationally representative sample of mother-child dyads in Lebanon in 2011–2012.

## Methods

The study was designed as a cross-sectional survey as part of the “Early Life Nutrition and Health in Lebanon (ELNAHL)” project [14].

### Sampling strategy

A representative sample of children (*N* = 1194) aged 5 years or younger of both sexes was drawn from households using a stratified cluster sampling design. The strata were the six Lebanese governorates and the clusters were selected at the district level. Within each district, households were selected following a probability proportional to size approach, whereby a higher number of participating households was drawn from more populous districts. Housing units constituted the primary sampling unit in the districts of Lebanon.

### Eligibility criteria

Mother-child dyads were eligible to participate in the study if the mother was Lebanese, aged 19–40 years, did not have hypertension or diabetes, and was not taking medications that interfere with eating and breastfeeding or affect body weight. Children were eligible if they were five years old or younger, were born at term (gestational age between 37 and 42 weeks), and had no chronic medical conditions, inborn errors of metabolism, or physical malformations that interfere with feeding patterns and body composition.

### Data collection

Data were collected between September 2011 and August 2012. Trained nutritionists administered the survey through face-to-face interviews with the mothers. The survey inquired about sociodemographic characteristics of the household, current and future family planning practices of the mother, access to maternal and child health services, and mother’s knowledge and practices related to infant feeding. Questions on infant feeding practices focused on the duration of total and exclusive breastfeeding, which was assessed using the life-long approach [15], the age of introduction of formula milk and solid food, and the reasons for breastfeeding, not breastfeeding, and breastfeeding cessation. Anthropometric measurements of the mother and child were collected. This study was approved by the Institutional Review Boards at the American University of Beirut (NUT.LN.13) and Purdue University (Protocol number: 1902021663). Mothers provided written informed consents.

### Definitions of infant feeding practices

Infant feeding practices were defined as follows:

1. Exclusive breastfeeding (EBF): The infant received breast milk from his/her mother or expressed breast milk and no other fluids or solids.
2. Mixed feeding: The infant received breast milk with formula milk and/or other fluids and/or solid food.
3. Exclusive bottle feeding (EBOT): The infant received formula milk with or without other fluids.
4. Bottle and solid feeding (BOT+SF): The infant received formula milk and/or other fluids and solid food.

### Statistical analysis

Frequencies with percentages (%) and means with standard deviations (SD) were calculated to describe categorical and continuous variables, respectively. Since only 17 mothers (1.8%) of the sample were underweight (Body Mass Index [BMI] < 18.5 kg/m^2^) at the time of the interview, they were merged with the normal weight mothers (BMI = 18.5–24.9 kg/m^2^) into one BMI category (normal weight <  25 kg/m^2^). Chi-squared test and Analysis of Variance (ANOVA) followed by post hoc comparisons (Bonferroni) were calculated, as appropriate, to compare infant feeding practices at 40 days and at 6 months by sociodemographic, maternal, and child characteristics. Multinomial logistic regression models were computed to estimate the adjusted odds ratio (AOR) and 95% confidence intervals (CI) of EBF compared to mixed feeding and EBOT at 40 days, as well as the odds of EBF compared to mixed feeding and BOT+SF at 6 months. Variables selected for inclusion in the multinomial analysis had a *p -*value < 0.10 in the bivariate analysis. All statistical analysis was conducted using the Statistical Analysis Package for Social Sciences (SPSS, version 24.0).

## Results

Of the 1194 eligible mother-child dyads that were contacted, 1029 participated in the survey (response rate 86%). Twenty-four surveys with missing data were excluded, leaving 1005 mother-child dyads in the analysis. At the time of the interview, 947 children were 40 days or older and had complete information about feeding practices at 40 days, while 893 children were 6 months or older and had complete information about feeding practices at 6 months and thereby included in the analysis at 40 days and at 6 months, respectively.

### Prevalence of exclusive breastfeeding and other infant feeding practices at 40 days and at 6 months

**Fig. 1:**
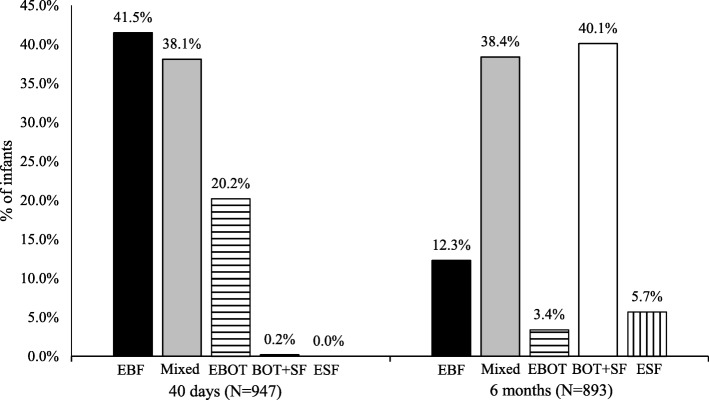
Prevalence of infant feeding practices at 40 days and 6 months in Lebanon. EBF: Exclusive breastfeeding; EBOT: Exclusive bottle feeding; BOT+SF: Bottle and solid feeding; ESF: Exclusive solid feeding

The prevalence of infant feeding practices at 40 days and at 6 months are presented in Fig. 1. Of the total 1005 children included in the analysis, 89.5% were ever breastfed. Among those, 27.8% were first breastfed within 1 h after delivery, 53.1% after 1 h but within the first 24 h, and 19.1% within days after delivery (data not shown). At 40 days, 41.5% (95% CI 38.4, 44.7) of the 947 children were EBF, 38.1% (95% CI 34.8, 41.2) were mixed fed, and 20.2% (95% CI 17.5, 22.7) were EBOT. At 6 months, 12.3% (95% CI 10.2, 14.6) of the 893 children were EBF, 38.4% (95% CI 35.3, 41.7) were mixed fed, and 40.1% (95% CI 36.7, 43.3) were BOT+SF.

### Sociodemographic, maternal, and child characteristics by infant feeding practice at 40 days and at 6 months

Sociodemographic, maternal, and child characteristics by infant feeding practice at 40 days and at 6 months are presented in Table 1. There were significant differences between the infant feeding groups at 40 days in terms of paternal educational level, number of owned cars, number of children in the family, mode of delivery and child’s age, weight and height at the time of the interview (overall *p* - value < 0.05). Specifically, a lower proportion of EBOT children had fathers with an education at an intermediate level or lower compared to mixed fed children (Bonferroni adjusted *p* - value = 0.008 [data not shown]). A higher proportion of EBOT children were in families that owned two or more cars (*p* – value = 0.013) and had less than three children (*p* - value < 0.001) compared to EBF children. In addition, a higher proportion of EBOT children was delivered via Caesarian section (C-section) compared to EBF (*p* - value < 0.001) and mixed fed (*p* – value = 0.002) children. Exclusive breastfed children were older and taller at the time of the interview than mixed fed (*p* – value = 0.001 and 0.002, respectively) and EBOT (*p* - value = 0.002 and 0.016, respectively) children. Moreover, EBF children were heavier at the time of the interview than mixed fed children (*p* – value = 0.015).

**Table 1:** Sociodemographic, maternal, and child characteristics by infant feeding practice at 40 days and at 6 months in Lebanon

Characteristics ^**#**^	At 40 days (***N*** = 947)				At 6 months (***N*** = 893)			
	EBF (***n*** = 393)	Mixed fed (***n*** = 361)	EBOT (***n*** = 191)	***p*** - value*	EBF (***n*** = 110)	Mixed fed (***n*** = 343)	BOT + SF (***n*** = 358)	***p*** - value*
**Sociodemographic characteristics**								
Governorate of residence								
Beirut	43 (10.9)	31 (8.6)	15 (7.9)	0.074	14 (12.7) ^a,b^	31 (9.0) ^a^	33 (9.2) ^b^	**0.009**
Mount Lebanon	123 (31.3)	95 (26.3)	67 (35.1)		36 (32.7) ^a,b^	83 (24.2) ^a^	126 (35.2) ^b^	
North Lebanon	151 (38.4)	134 (37.1)	58 (30.4)		37 (33.6) ^a,b^	152 (44.3) ^a^	107 (29.9) ^b^	
South Lebanon & Nabatieh	53 (13.5)	67 (18.6)	34 (17.8)		17 (15.5) ^a,b^	52 (15.2) ^a^	64 (17.9) ^b^	
Bekaa	23 (5.9)	34 (9.4)	17 (8.9)		6 (5.5) ^a,b^	25 (7.3) ^a^	28 (7.8) ^b^	
Family monthly income (USD)								
≤ 400	39 (9.9)	38 (10.6)	10 (5.2)	0.341	6 (5.5) ^a,b^	49 (14.4) ^a^	17 (4.8) ^b^	**< 0.001**
400.1–1000	180 (45.9)	153 (42.6)	89 (46.6)		56 (50.9) ^a,b^	161 (47.2) ^a^	157 (44.0) ^b^	
1000.1–2000	66 (16.8)	65 (18.1)	47 (24.6)		15 (13.6) ^a,b^	49 (14.4) ^a^	82 (23.0) ^b^	
> 2000	36 (9.2)	30 (8.4)	15 (7.9)		13 (11.8) ^a,b^	16 (4.7) ^a^	40 (11.2) ^b^	
Doesn’t know	42 (10.7)	42 (11.7)	16 (8.4)		12 (10.9) ^a,b^	44 (12.9) ^a^	29 (8.1) ^b^	
Refused to answer	29 (7.4)	31 (8.6)	14 (7.3)		8 (7.3) ^a,b^	22 (6.5) ^a^	32 (9.0) ^b^	
Paternal educational level								
Intermediate or lower	210 (54.7) ^a,b^	205 (57.6) ^a^	92 (48.2) ^b^	**0.035**	57 (52.3) ^a,b^	208 (61.7) ^a^	175 (49.2) ^b^	**0.023**
Secondary education or technical diploma	113 (29.4) ^a,b^	96 (27.0) ^a^	76 (39.8) ^b^		35 (32.1) ^a,b^	87 (25.8) ^a^	123 (34.6) ^b^	
University degree	61 (15.9) ^a,b^	55 (15.4) ^a^	23 (12.0) ^b^		17 (15.6) ^a,b^	42 (12.5) ^a^	58 (16.3) ^b^	
Paternal employment status								
Government or private sector	229 (58.3)	209 (57.9)	117 (61.3)	0.400	67 (60.9)	196 (57.1)	219 (61.2)	0.414
Self-employed	137 (34.9)	136 (37.7)	67 (35.1)		34 (30.9)	130 (37.9)	122 (34.1)	
Unemployed	27 (6.9)	16 (4.4)	7 (3.7)		9 (8.2)	17 (5.0)	17 (4.7)	
House ownership								
Yes	230 (58.5)	215 (59.6)	122 (63.9)	0.454	60 (54.5)	206 (60.1)	222 (62.0)	0.375
No	163 (41.5)	146 (40.4)	69 (36.1)		50 (45.5)	137 (39.9)	136 (38.0)	
Number of owned cars								
0	90 (22.9) ^a^	87 (24.1) ^a,b^	36 (18.8) ^b^	**0.046**	24 (21.8) ^a,b^	96 (28.0) ^a^	57 (15.9) ^b^	**< 0.001**
1	235 (59.8) ^a^	204 (56.5) ^a,b^	102 (53.4) ^b^		66 (60.0) ^a,b^	202 (58.9) ^a^	198 (55.3) ^b^	
≥ 2	68 (17.3) ^a^	70 (19.4) ^a,b^	53 (27.7) ^b^		20 (18.2) ^a,b^	45 (13.1) ^a^	103 (28.8) ^b^	
Crowding index (individuals/room)								
≥ 1	349 (88.8)	309 (85.6)	163 (85.3)	0.334	99 (90.0)	303 (88.3)	301 (84.1)	0.138
< 1	44 (11.2)	52 (14.4)	28 (14.7)		11 (10.0)	40 (11.7)	57 (15.9)	
Number of children								
1–2	199 (50.6) ^a^	203 (56.2) ^a,b^	127 (66.5) ^b^	**0.001**	59 (53.6) ^a,b^	162 (47.2) ^a^	229 (64.0) ^b^	**< 0.001**
≥ 3	194 (49.4) ^a^	158 (43.8) ^a,b^	64 (33.5) ^b^		51 (46.4) ^a,b^	181 (52.8) ^a^	129 (36.0) ^b^	
**Maternal characteristics**								
Age (years) ^$^	32.1 ± 5.7	31.2 ± 6.6	31.4 ± 6.6	0.122	32.5 ± 5.9	32.2 ± 6.3	31.3 ± 6.4	0.064
Educational level								
Intermediate or lower	201 (51.1)	180 (49.9)	79 (41.4)	0.067	52 (47.3) ^a,b^	193 (56.3) ^a^	150 (41.9) ^b^	**0.001**
Secondary education or technical diploma	103 (26.2)	106 (29.4)	72 (37.7)		30 (27.3) ^a,b^	97 (28.3) ^a^	121 (33.8) ^b^	
University degree	89 (22.6)	75 (20.8)	40 (20.9)		28 (25.5) ^a,b^	53 (15.5) ^a^	87 (24.3) ^b^	
Employment status								
Employed	65 (16.5)	62 (17.2)	40 (20.9)	0.404	14 (12.7)	47 (13.7)	71 (19.8)	0.050
Housewives	328 (83.5)	299 (82.8)	151 (79.1)		96 (87.3)	296 (86.3)	287 (80.2)	
BMI (kg/m^2^)								
Normal weight (< 25)	163 (44.4)	137 (42.2)	71 (40.3)	0.903	57 (53.3) ^a^	118 (37.7) ^b^	148 (45.8) ^a,b^	**0.024**
Overweight (25–29.99)	117 (31.9)	108 (33.2)	58 (33.0)		29 (27.1) ^a^	103 (32.9) ^b^	105 (32.5) ^a,b^	
Obese (≥ 30)	87 (23.7)	80 (24.6)	47 (26.7)		21 (19.6) ^a^	92 (29.4) ^b^	70 (21.7) ^a,b^	
**Child characteristics**								
Age (months) ^$^	29.6 ± 15.6 ^a^	25.9 ± 16.1 ^b^	25.2 ± 16.4 ^b^	**0.001**	32.3 ± 13.7 ^a^	28.9 ± 14.7 ^a,b^	27.5 ± 15.5 ^b^	**0.014**
Gender								
Male	187 (47.6)	192 (53.2)	103 (53.9)	0.204	49 (44.5)	174 (50.7)	195 (54.5)	0.176
Female	206 (52.4)	169 (46.8)	88 (46.1)		61 (55.5)	169 (49.3)	163 (45.5)	
Mode of delivery								
Vaginal	241 (61.5) ^a^	197 (55.0) ^a^	79 (41.4) ^b^	**< 0.001**	74 (67.3) ^a^	205 (59.9) ^a^	157 (44.1) ^b^	**< 0.001**
C-section	151 (38.5) ^a^	161 (45.0) ^a^	112 (58.6) ^b^		36 (32.7) ^a^	137 (40.1) ^a^	199 (55.9) ^b^	
Birthweight (g) ^$^	3218.2 ± 500.9	3244.4 ± 562.7	3169.7 ± 547.0	0.297	3293.5 ± 512.0	3232.1 ± 540.3	3188.6 ± 536.9	0.178
Birth length (cm) ^$^	50.3 ± 3.0	50.3 ± 3.0	50.3 ± 3.4	0.986	50.3 ± 3.3	50.3 ± 2.8	50.3 ± 3.1	0.988
Weight (kg) ^$^	13.2 ± 3.8 ^a^	12.5 ± 4.2 ^b^	12.7 ± 3.9 ^a,b^	**0.043**	14.0 ± 3.5 ^a^	12.9 ± 3.5 ^b^	13.2 ± 3.5 ^a,b^	**0.018**
Height (cm) ^$^	87.9 ± 12.8 ^a^	84.8 ± 14.2 ^b^	84.9 ± 14.3 ^b^	**0.004**	90.6 ± 11.1 ^a^	87.4 ± 12.0 ^b^	87.4 ± 12.4 ^b^	**0.035**

^#^ Data are presented as frequency (%) unless otherwise stated

^$^ Data are presented as mean ± SD

* Overall *p*-values compare the three infant feeding groups together and are calculated using chi-squared test for categorical variables and ANOVA for continuous variables

^a-b^ Infant feeding practices with different superscripts differ significantly after Bonferroni adjustment (*p* - value < 0.0167)

At 6 months, there were significant differences between the infant feeding groups in terms of governorate of residence, paternal and maternal education, monthly income, number of owned cars, and number of children in the family (overall *p* - value < 0.05). The groups also differed in terms of maternal BMI, mode of delivery, and child’s age, weight and height at the time of the interview. A lower proportion of BOT+SF children were in families that lived in North Lebanon (Bonferroni adjusted *p* – value = 0.001), had lower monthly income (*p* - value < 0.001), and had fathers and mothers with an education at an intermediate level or lower (*p* – value = 0.004 and <  0.001, respectively) compared to mixed fed children. In addition, a higher proportion of BOT+SF children were in families that owned two or more cars (*p* - value < 0.001) and had less than three children (*p* - value < 0.001) compared to mixed fed children. A higher proportion of EBF children had mothers with normal weight compared to mixed fed children (*p* – value = 0.016). A higher proportion of BOT+SF children was delivered via C-section compared to EBF (*p* - value < 0.001) and mixed fed (*p* - value < 0.001) children. EBF children were older than BOT+SF (*p* – value = 0.002), heavier than mixed fed children (*p*-value = 0.005), and taller than mixed fed (*p* - value = 0.013) and BOT+SF (*p* – value = 0.011) children.

Table 2 presents the results of the multinomial logistic regression analysis of the factors associated with EBF for 40 days. Although certain variables had significant overall and/or Bonferroni adjusted *p*-values in the bivariate analysis, they were not significant after adjusting for all the variables in the model. Children in families owning two or more cars and those delivered via C-section had lower odds of EBF than EBOT at 40 days postpartum (AOR 0.45, 95% CI 0.24, 0.83 and AOR 0.49, 95% CI 0.34, 0.71, respectively). On the other hand, children in families with three or more children had higher odds of EBF than EBOT at 40 days (AOR 1.76, 95% CI 1.19, 2.60).

**Table 2:** Multinomial logistic regression analysis of factors associated with exclusive breastfeeding for 40 days in Lebanon

Characteristics	At 40 days (***N*** = 947) ^**a**^	
	EBF vs Mixed feeding ^**1**^	EBF vs EBOT ^**2**^
**Sociodemographic characteristics**		
Governorate of residence		
Beirut	1.00	1.00
Mount Lebanon	1.04 (0.59, 1.83)	0.89 (0.44, 1.79)
North Lebanon	0.88 (0.51, 1.54)	1.00 (0.49, 2.02)
South Lebanon & Nabatieh	0.60 (0.33, 1.10)	0.64 (0.30, 1.38)
Bekaa	0.54 (0.26, 1.14)	0.58 (0.23, 1.43)
Paternal educational level		
Intermediate or lower	1.00	1.00
Secondary education or technical diploma	1.11 (0.66, 1.86)	1.44 (0.75, 2.76)
University degree	1.25 (0.85, 1.82)	0.86 (0.55, 1.34)
Number of owned cars		
0	1.00	1.00
1	1.10 (0.75, 1.60)	0.93 (0.57, 1.52)
≥ 2	0.84 (0.50, 1.41)	**0.45 (0.24, 0.83)**
Number of children		
1–2	1.00	1.00
≥ 3	1.23 (0.90, 1.68)	**1.76 (1.19, 2.60)**
**Maternal characteristics**		
Educational level		
Intermediate or lower	1.00	1.00
Secondary education or technical diploma	1.09 (0.67, 1.76)	1.39 (0.77, 2.49)
University degree	0.85 (0.58, 1.24)	0.76 (0.48, 1.19)
**Child characteristics**		
Age (months)	1.00 (0.98, 1.03)	1.03 (0.99, 1.06)
Mode of delivery		
Vaginal	1.00	1.00
C-section	0.78 (0.58, 1.06)	**0.49 (0.34, 0.71)**
Weight (kg)	0.96 (0.87, 1.07)	0.93 (0.83, 1.05)
Height (cm)	1.03 (0.98, 1.07)	1.01 (0.95, 1.06)

^**a**^ Data are presented as adjusted odds ratio (95% confidence interval)

^1^ The reference category is Mixed feeding; ^2^ The reference category is EBOT

*EBF* Exclusive breastfeeding; *EBOT* Exclusive bottle feeding

Table 3 presents the results of the multinomial logistic regression analysis at 6 months. The odds of EBF were half those of mixed feeding at 6 months among children of overweight (AOR 0.50, 95% CI 0.26, 0.95) or obese (AOR 0.56, 95% CI 0.32, 0.98) mothers. Children in families owning two or more cars and those delivered via C-section had lower odds of EBF than of BOT+SF at 6 months (AOR 0.32, 95% CI 0.14, 0.77 and AOR 0.39, 95% CI 0.24, 0.65, respectively).

**Table 3:** Multinomial logistic regression analysis of factors associated with exclusive breastfeeding at 6 months in Lebanon

Characteristics	At 6 months (***N*** = 893) ^**a**^	
	EBF vs Mixed feeding ^**1**^	EBF vs BOT + SF ^**2**^
**Sociodemographic characteristics**		
Governorate of residence		
Beirut	1.00	1.00
Mount Lebanon	1.24 (0.55, 2.80)	0.81 (0.36, 1.82)
North Lebanon	0.73 (0.32, 1.70)	0.74 (0.32, 1.72)
South Lebanon & Nabatieh	1.09 (0.44, 2.74)	0.73 (0.29, 1.81)
Bekaa	0.77 (0.23, 2.59)	0.56 (0.17, 1.85)
Family monthly income (USD)		
≤ 400	1.00	1.00
400.1–1000	0.39 (0.11, 1.47)	2.08 (0.51, 8.50)
1000.1–2000	1.19 (0.47, 3.02)	1.98 (0.80, 4.88)
> 2000	0.75 (0.25, 2.23)	0.87 (0.31, 2.45)
Doesn’t know	1.47 (0.44, 4.93)	1.86 (0.60, 5.75)
Refused to answer	1.08 (0.35, 3.27)	2.32 (0.77, 7.00)
Paternal educational level		
Intermediate or lower	1.00	1.00
Secondary education or technical diploma	0.88 (0.37, 2.12)	1.02 (0.43, 2.40)
University degree	1.16 (0.65, 2.09)	0.97 (0.54, 1.74)
Number of owned cars		
0	1.00	1.00
1	0.93 (0.51, 1.68)	0.66 (0.35, 1.23)
≥ 2	1.01 (0.42, 2.43)	**0.32 (0.14, 0.77)**
Number of children		
1–2	1.00	1.00
≥ 3	0.85 (0.50, 1.45)	1.18 (0.69, 2.02)
**Maternal characteristics**		
Age (years)	1.01 (0.97, 1.05)	1.03 (0.98, 1.07)
Educational level		
Intermediate or lower	1.00	1.00
Secondary education or technical diploma	1.46 (0.66, 3.24)	1.73 (0.80, 3.78)
University degree	0.89 (0.48, 1.64)	1.03 (0.56, 1.90)
Employment status		
Employed	1.00	1.00
Housewives	1.70 (0.82, 3.53)	1.63 (0.80, 3.30)
BMI (kg/m^2^)		
Normal weight (< 25)	1.00	1.00
Overweight (25–29.99)	**0.50 (0.26, 0.95)**	0.70 (0.36, 1.35)
Obese (≥ 30)	**0.56 (0.32, 0.98)**	0.60 (0.34, 1.05)
**Child characteristics**		
Age (months)	0.99 (0.95, 1.04)	1.01 (0.96, 1.05)
Mode of delivery		
Vaginal	1.00	1.00
C-section	0.67 (0.40, 1.11)	**0.39 (0.24, 0.65)**
Weight (kg)	1.17 (0.98, 1.39)	1.06 (0.90, 1.26)
Height (cm)	1.00 (0.92, 1.08)	1.00 (0.92, 1.08)

^**a**^ Data are presented as adjusted odds ratio (95% confidence interval)

^1^ The reference category is Mixed feeding; ^2^ The reference category is BOT+SF

*EBF* Exclusive breastfeeding; *BOT+SF* Bottle and solid feeding

## Discussion

To our knowledge, this study is the first in the Middle East to examine the prevalence and predictors of EBF during the 40-day rest period. From a nationally representative survey in Lebanon, we report a prevalence of EBF of 41.5% at 40 days and 12. 3% at 6 months.

While belonging to a family with more children was positively associated with EBF at 40 days, belonging to a family owning more cars or being born via C-section was associated with lower odds of EBF at 40 days and at 6 months. In addition, the odds of EBF for 6 months were lower among children whose mothers were overweight or obese.

The 40-day rest period is practiced in several cultures worldwide including the Amazon [16], Greece [4], China [17], Malaysia [18], Nepal [6], India [5], Burma [19], Turkey [20], Negev Bedouins [1, 3], and Egypt [2]. However, few studies have examined the prevalence of total and exclusive breastfeeding during this period [1, 2]. In a study of Negev Bedouin Arab women [1], 24% of the women exclusively breastfed their infants for the first 2 months regardless of whether women received help during the first 40 days. In a qualitative study of maternal beliefs about breastfeeding in a poor urban neighborhood in Egypt [2], women reported exclusively breastfeeding their infants for the first 40 days, after which they supplemented the breastmilk with fluids and foods to promote growth and fatness and to decrease the time spent breastfeeding. In Lebanon, the prevalence of EBF for 8–12 weeks was 27.4% among a sample of first-time mothers residing in the capital, Beirut, as part of a randomized trial of postpartum depression [21]. The lower rate of EBF in that study compared to ours (41.5%) might be due to the longer duration at which EBF was assessed (56–84 days), the sample of women representing an urban population in which EBF rates have been suggested to be lower [22, 23], and the selective nature of participating in a postpartum depression study.

The prevalence of EBF for 6 months in our study was estimated at 12.3% in 2012. The figure is slightly higher than that estimated by Batal et al. in 2004 (10.1%) among a national sample of women recruited from health centers operated by the Ministry of Social Affairs [11]. A more recent study by Mattar et al. in 2016 estimated the prevalence of EBF for 4–6 months at 16.5% among mothers with 12–36-month-old toddlers recruited from a representative sample of licensed daycare centers by the Lebanese Ministry of Public Health [12]. Compared to other countries in the Middle East, our prevalence estimate of EBF for 6 months is closest to that of 12.2% in Saudi Arabia [24]. Despite the fact that the Levant countries are in geographic proximity and share similar traditions and population characteristics, the rates of EBF for 6 months in Lebanon were only similar to those in Syria at 12.9% [25], but lower than those in the Gaza Strip in Palestine (24.4%) [26] and higher than those in Jordan (1%) [27].

A higher number of cars owned in a household, an indicator of higher socio-economic status, was associated with lower odds of EBF at 40 days and at 6 months. Indeed, the number of cars was correlated with other socio-economic variables including mother’s and father’s educational level, family monthly income, house ownership, and crowding index (data not shown). Unlike in high-income countries where breastfeeding rates are higher among wealthier and more educated women, breastfeeding rates are lower and the duration is shorter among wealthier women in low- and middle-income countries [28]. According to two cross-sectional studies conducted in middle-income countries, the first in Nigeria in 2012 [29] and the second in Morocco in 2016 [30], mothers of higher socio-economic status had a lower likelihood of EBF for 6 months. Lebanon, a middle-income country, followed a similar trend.

Compared to vaginal delivery, C-section delivery was consistently associated with lower odds of EBF at 40 days and at 6 months. Other studies from Lebanon yielded mixed results on the association between mode of delivery and exclusive breastfeeding. While Mattar et al. [12] found that C-section delivery was associated with shorter duration of exclusive breastfeeding, Batal et al. [11] did not. However, the latter found differences in hospital practices that support or hinder breastfeeding initiation, which in turn affect breastfeeding exclusivity and duration [31]. For example, a smaller proportion of women who delivered via C-section reported that the hospitals discussed the benefits of breastfeeding with them, allowed 24-h rooming-in, and brought the baby for night feeds compared to women who delivered vaginally. Indeed, 24-h rooming-in and bringing the baby often to the mother for feeding were associated with higher odds of breastfeeding initiation within few hours after birth. Rates of C-section delivery are high in Lebanon with an estimated prevalence of 49% among a sample of women who delivered between 2000 and 2015 [32]. This prevalence greatly exceeds the rate for C-section deliveries of 10–15% suggested by the WHO [33]. Caesarian section delivery along with the associated maternal and newborn complications have been reported to hinder skin-to-skin contact after birth and delay breastfeeding initiation which in turn reduce breastfeeding duration [34, 35].

The number of children in the family was associated with EBF for 40 days, where mothers with a larger number of children were more likely to EBF. In a prospective cohort study conducted in six low- and middle-income countries in 2010 [36], nulliparity was associated with lower odds of EBF for the first 42 days postpartum in two of the included countries, Guatemala and Pakistan.

Overweight and obese mothers were less likely to EBF for 6 months. Given the cross-sectional nature of the study, we cannot determine the direction of the association, notably whether mothers who were presumably overweight or obese in pregnancy were less likely to EBF or whether mothers who did not EBF were more likely to be overweight or obese at the time of the interview. Evidence from the literature supports both directions of association between maternal BMI and duration of EBF. In a cross-sectional study in Germany [37], EBF duration was shorter among obese mothers than among normal-weight mothers. In an analysis of CDC Pregnancy Risk Assessment Monitoring System of 19,145 mothers from 2004 to 2008 [38], overweight and obese mothers, compared to the normal weight, had higher odds of discontinuing breastfeeding before 6 months due to insufficient milk and breastfeeding difficulties. On the other hand, shorter breastfeeding duration has been implicated in maternal overweight and obesity. In a study of 212 women in Finland in 2007 who were surveyed 16–20 years postpartum [39], mothers who breastfed for less than 6 months had higher total body fat mass and fat mass percentage than mothers who breastfed for more than 6 months.

This study has a number of limitations. First, it is a cross-sectional study with unclear chronology of the factors associated with exclusive breastfeeding. Second, mothers reported infant feeding practices of their children, with potential for more recall bias among mothers of older children due to a longer time since delivery; however, almost half of the children were younger than 2 years of age.

## Conclusions

The study offers a unique lens into infant feeding practices related to the 40-day rest period and first 6 months of life in Lebanon. The prevalence of EBF at 40 days and at 6 months was 41.5 and 12.3%, respectively. The inverse association of higher socio-economic status, as reflected by the number of cars owned, and C-section delivery with lower odds of EBF persisted across the first 6 months. Future research should investigate the factors associated with EBF in prospective cohort studies and help to better understand the cultural practice of the 40-day rest period in relation to breastfeeding. Such research can guide effective planning for interventions to improve breastfeeding practices and ultimately children’s health status.

## Data Availability

The dataset analyzed during the current study are available from the corresponding author on reasonable request.

## **Abbreviations**

EBF: Exclusive breastfeeding
EBOT: Exclusive bottle feeding
BOT+SF: Bottle and solid feeding
ESF: Exclusive solid feeding
ANOVA: Analysis of variance
AOR: Adjusted odds ratio
CI: Confidence interval
BMI: Body mass index
